# Supplementary material for: A UMLS-based spell checker for natural language processing in vaccine safety
Source: BMC Med Inform Decis Mak. 2007 Feb 12;7:3. doi: 10.1186/1472-6947-7-3 (PMC1805499; doi:10.1186/1472-6947-7-3)
Supplement: Additional file 13 — RAP application directory. Sets up directory for RAP (RDF application for PHP) source code [file 1472-6947-7-3-S13.gz › rap/doc/phpdoc/__filesource/fsource_ontModel__ontModelOntModelP.php.html]

xml version="1.0" encoding="iso-8859-1"?


File Source for OntModelP.php


# Source for file OntModelP.php

Documentation is available at OntModelP.php

```
1. <?PHP
2. /**
3. * ----------------------------------------------------------------------------------
4. * OntModel Package
5. * ----------------------------------------------------------------------------------
6. *
7. * Description               : OntModel package
8. *
9. * History:
10. * 10-04-2004                : Initial version.
11. *
12. * Author: Daniel Westphal    <mail at d-westphal dot de>
13. * @package ontModel
14. *  ----------------------------------------------------------------------------------
15. */
16. // Include OntModel classes
17. include_once( RDFAPI_INCLUDE_DIR . PACKAGE_RESMODEL);
18. require_once( RDFAPI_INCLUDE_DIR . 'ontModel/OntVocabulary.php');
19. require_once( RDFAPI_INCLUDE_DIR . 'ontModel/RdfsVocabulary.php');
20. require_once( RDFAPI_INCLUDE_DIR . 'ontModel/OntModel.php');
21. require_once( RDFAPI_INCLUDE_DIR . 'ontModel/OntResource.php');
22. require_once( RDFAPI_INCLUDE_DIR . 'ontModel/OntProperty.php');
23. require_once( RDFAPI_INCLUDE_DIR . 'ontModel/OntClass.php');
24. require_once( RDFAPI_INCLUDE_DIR . 'ontModel/Individual.php');

26. ?>
```

Documentation generated on Thu, 7 Jul 2005 13:42:23 +0200 by phpDocumentor 1.3.0RC3
